# *Polychromophilus* spp. (Haemosporida) in Malagasy bats: host specificity and insights on invertebrate vectors

**DOI:** 10.1186/s12936-018-2461-8

**Published:** 2018-08-31

**Authors:** Beza Ramasindrazana, Steven M. Goodman, Najla Dsouli, Yann Gomard, Erwan Lagadec, Milijaona Randrianarivelojosia, Koussay Dellagi, Pablo Tortosa

**Affiliations:** 1 UMR PIMIT “Processus Infectieux en Milieu Insulaire Tropical”, INSERM 1187, CNRS 9192, IRD 249, Plateforme de Recherche CYROI, Université de La Réunion, 97490 Sainte Clotilde, La Réunion France; 2 Association Vahatra, BP 3972, 101 Antananarivo, Madagascar; 3 Institut Pasteur de Madagascar, BP 1274, Ambatofotsikely, 101 Antananarivo, Madagascar; 4 The Field Museum of Natural History, Chicago, IL 60605 USA; 5 Faculté des Sciences, Université de Toliara, 601 Toliara, Madagascar; 6 Present Address: Institut Pasteur (International Division), 25-28 Rue du Dr Roux, 75015 Paris, France

**Keywords:** *Polychromophilus*, Bats, Nycteribiidae, Phylogeny, Vectors, Madagascar

## Abstract

**Background:**

Bats are home to diverse haemosporidian parasites namely *Plasmodium* and *Plasmodium*-related. While information is available at a worldwide level, haemosporidian infection in bats from Madagascar is still scarce and recent changes in the taxonomy of the island’s bat fauna, particularly the description of several new species, require a reassessment of previously described patterns, including blood parasite ecology and vectorial transmission.

**Methods:**

A sample representing seven of the nine known bat families and 31 of the 46 currently recognized taxa from Madagascar and collected in the western and central portions of the island were screened by PCR for the presence of *Polychromophilus*. In addition, Nycteribiidae flies parasitizing Miniopteridae and Vespertilionidae were screened for parasites with the aim to better understand aspects of vector transmission. Phylogenetic reconstruction using the mitochondrial cytochrome *b* encoding gene was used in a Bayesian analysis to examine the relationship between *Polychromophilus* recovered from Malagasy bats and those identified elsewhere.

**Results:**

*Polychromophilus* infection was restricted to *Miniopterus* spp. (Miniopteridae), *Myotis goudoti* (Vespertilionidae), and *Paratriaenops furculus* (Rhinonycteridae), with an overall infection rate of 13.5%. *Polychromophilus melanipherus* was found infecting *Miniopterus* spp. and *P. furculus*, whereas *Polychromophilus murinus* was only recovered from *M. goudoti*. These two protozoan parasites species were also detected in bat flies species known to parasitize *Miniopterus* spp. and *M. goudoti*, respectively. Generalized linear model analyses were conducted to elucidate the effect of species and sex on haemoparasites infection in *Miniopterus* spp., which revealed that males have higher risk of infection than females and prevalence differed according to the considered *Miniopterus* host. Molecular screening of nycteribiid flies revealed three positive species for *Polychromophilus* spp., including *Penicillidia* sp. (cf. *fulvida*), *Penicillidia leptothrinax*, and *Nycteribia stylidiopsis*. These three fly species are known to parasitize *Miniopterus* spp. and *M. goudoti* and should be considered as potential vectors of *Polychromophilus* spp.

**Conclusion:**

Phylogenetic analyses demonstrated the existence of at least four distinct clades within the genus *Polychromophilus*, two of which were documented in the present study. The screening of nycteribiid flies overlaid on the highly diversified genus *Miniopterus*, provides considerable insight into parasite transmission, with bat infection being associated with their roosting behaviour and the occurrence of specific arthropod vectors.

**Electronic supplementary material:**

The online version of this article (10.1186/s12936-018-2461-8) contains supplementary material, which is available to authorized users.

## Background

A number of different studies have been carried out to understand the evolutionary biology of haemosporidian parasites [1, 2]. The group *Plasmodium*, which infects humans, has been examined in detail because of its public health consequences [3, 4]. The genus *Plasmodium* has also been documented in different vertebrate groups, such as reptiles, birds and mammals, including non-human primates, based on morphological and molecular studies [1, 5, 6]. In other groups of vertebrates, malaria-related parasites are also known and these parasites have a common characteristic in their life cycle in that arthropods act as vectors. Malaria and malaria-related parasites form a paraphyletic groups within the haemosporidian and understanding the existing diversity is a key to having greater insight into their evolutionary biology.

As far as bats are concerned, their longevity, gregarious behaviour, dispersal potential and possible permissive immunity system have been proposed to facilitate parasite infection and maintenance [7]. Bats are hosts to different malaria and malaria-related parasites [6, 8–11]. To date, seven bat families are known to be infected by haemosporidian parasites: Hipposideridae, Megadermatidae, Miniopteridae, Pteropodidae, Rhinolophidae, Rhinonycteridae and Vespertilionidae [6, 8, 10, 12]. Different morphological characters associated with the taxonomy of bat malarial parasites have been previously published [13–16], and their systematics as well as species diversity have been clarified through molecular approaches [6, 9, 17, 18]. Thus far, based on morphological and molecular studies, eight genera of haemosporidian parasites are recognized to infect bats: *Biguetiella*, *Dionisia*, *Hepatocystis*, *Johnsprentia*, *Nycteria*, *Plasmodium*, *Polychromophilus* and *Sprattiella* [6, 8, 10, 15, 16, 18, 19]. With regards to the genus *Polychromophilus*, two species, *Polychromophilus melanipherus* and *Polychromophilus murinus*, were originally described from *Miniopterus schreibersii* and *Vespertilio murinus*, respectively [20]. These two haemosporidian taxa are known to infect bats belonging to the Miniopteridae and Vespertilionidae in Africa and Europe [9, 17]. Further, previous morphological descriptions identified three additional species, namely *Polychromophilus deanei*, *Polychromophilus adami* and *Polychromophilus corradetti*, infecting *Myotis nigricans* [13], *Miniopterus minor* and *M. schreibersii*, respectively [12]. In the literature, parasites infecting *Myotis* spp., *Eptesicus fuscus* and *V. murinus* have been placed within the genus *Bioccala*, formerly recognized as a subgenus of *Polychromophilus* [16, 21]; however, this has yet to be verified based on molecular studies.

As far as bats from Madagascar are concerned, considerable progress has been made in recent years regarding their systematics and taxonomy, and the fauna is currently composed of 46 recognized species of which nearly 80% are endemic [22–26]. Aspects of their roosting ecology and distribution have also been studied [23, 27]. Research examining blood parasite diversity in Malagasy bats using blood smear screening focused on 14 different bat species [28] and found the presence of Haemoproteidae in *Miniopterus gleni* and *Miniopterus manavi* sensu lato (Miniopteridae), *Myotis goudoti* (Vespertilionidae) and *Paratriaenops furculus* (formerly placed in the genus *Triaenops*) (Rhinonycteridae). Further, Duval et al. [8], reported the presence of Haemosporidia in *M. manavi* s.l. and *M. goudoti* without details on the taxonomy of these parasites. However, recent morphological and molecular studies revealed that these hemosporidian parasites are from two distinct clusters within *Polychromophilus*: *P. melanipherus* and *P. murinus*, detected in *M. manavi* s.l. and *M. goudoti*, respectively [17]. It is important to note that the number of recognized species within the genus *Miniopterus* on Madagascar has increased from four [22] to 12 [23, 24, 26] and *M. manavi* cited by Peterson and colleagues [22] represents a complex of paraphyletic species composed of at least eight cryptic taxa [23, 24, 26]. Further, different sister species relationships have been described within Malagasy members of this genus [29].

These different scientific advancements underline that available information on haemosporidian parasites infecting bats from Madagascar is scarce. Further, details on infection rates and host identity require further investigation to understand parasite ecology and distribution across the island.

## Methods

### Sampling sites and techniques

**Fig. 1:**
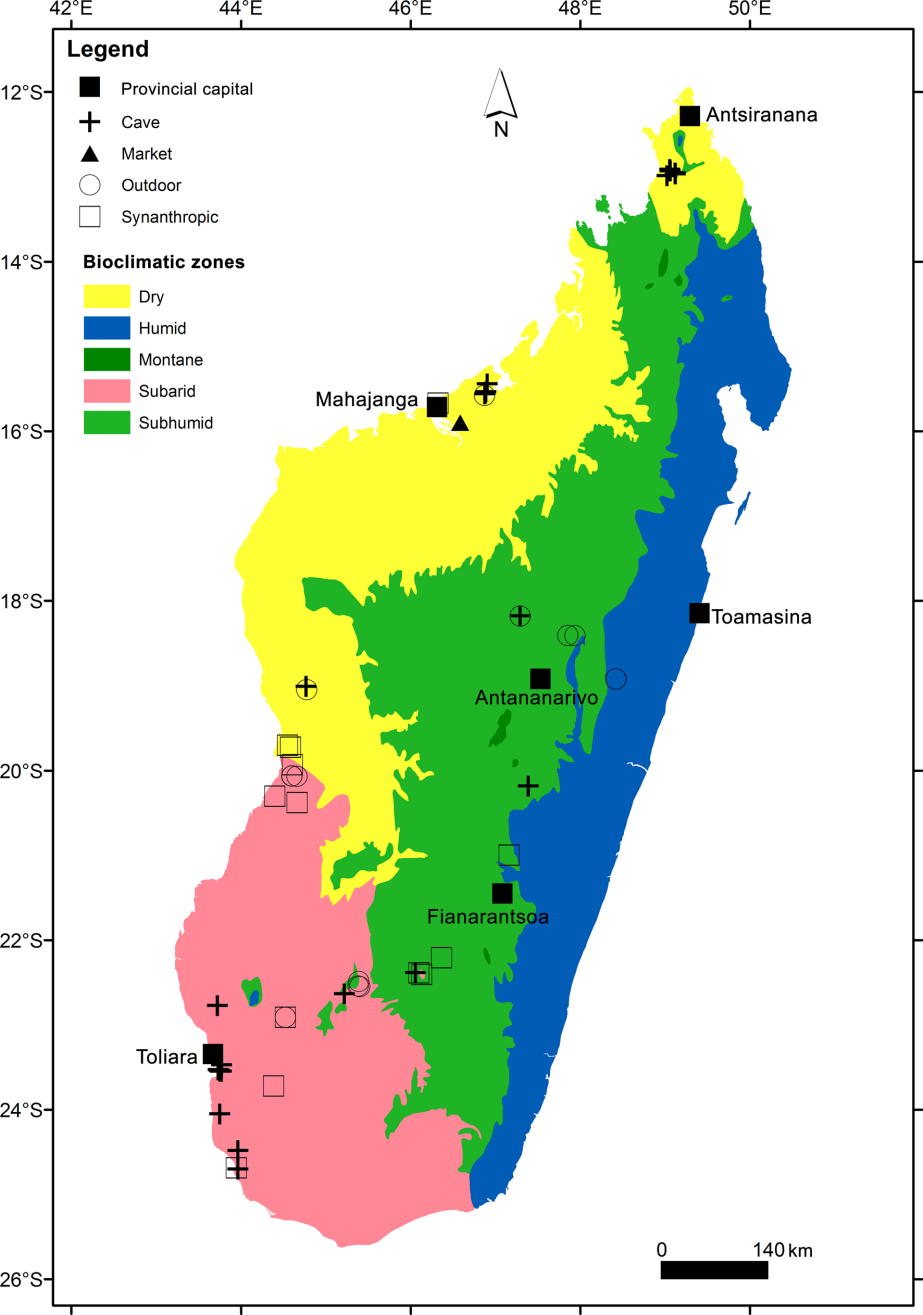
Localization map showing the different sites sampled across Madagascar in the present study and overlaid on different bioclimatic zones

Fifty-two sites were visited from February 2012 to March 2013 in different areas of Madagascar to sample bats associated with taxonomic studies [25], ecology and distribution [27], as well as hosted ectoparasite and microparasites [30–33]. Bats were captured using mist nets and harp traps, which were most often placed at cave entrances or across bat flight pathways (Fig. 1). In a few cases, a butterfly net was used to sample individuals from cave and synanthropic day-roost sites. The exception was for *Pteropus rufus*, a CITES Appendix II species, for which living individuals were purchased in a market and not physically captured by the field research group. This species is considered as bushmeat on Madagascar and exportation of tissue samples for scientific work needed a CITES permits (cf. Ethical approval and consent to participate).

Upon capture, each individual bat, excluding *Pteropus rufus* as noted above, was placed in a clean cloth bag. Further, Nycteribiidae flies, an obligate parasite of bats [34] and parasitizing members of the families Miniopteridae and Vespertilionidae, were directly collected from individual bats for morphological and molecular identification [30, 33]. Voucher specimens were deposited at the Université d’Antananarivo, Mention Zoologie et Biodiversité Animale (formerly Département de Biologie Animale) (UADBA), Antananarivo, and at the Field Museum of Natural History (FMNH), Chicago.

### DNA extraction and PCR screening

A pool of approximately 1 mm^3^ of kidney, lung and spleen from each individual bat was crushed in DMEM medium using two 3 mm-tungsten beads in a Tissue Lyser II (Qiagen, Valencia, CA). Subsequently, the mixture was centrifuged and the supernatant used for nucleic acids extraction. Total nucleic acids were extracted using EZ1 robot, with the viral mini kit v2.0 according to the manufacturer’s protocol (Qiagen Valencia, CA, USA), which has been routinely used in the PIMIT laboratory for the screening of both RNA and DNA parasites as previously described [32, 35, 36]. Nycteribiidae recovered from bats belonging to the families Miniopteridae and Vespertilionidae were crushed in a Tissue LyserII using two 3 mm tungsten beads, and total nucleic acids were extracted with the Qiagen biorobot with the viral mini kit v2.0 following manufacturer’s protocol (Qiagen Valencia, CA, USA) (see [30] for additional information).

Bat samples and Nycteribiidae flies were screened for the presence of *Polychromophilus* spp. using a previously described nested PCR protocol targeting the mitochondrial *cytochrome b* locus (*cyt b*) of haemosporidian parasites, which has been used to screen bats from Madagascar, Cambodia and Switzerland [8, 17, 18]. Primary PCR were conducted in a 25 µl reaction volume containing 12.5 µl of GoTaq^®^ Hot start green master mix (Promega, Madison, WI, USA), 1 µl of each Plas1/Plas2 primer at 0.4 µM and 1 µl of total nucleic acid as template. The balance of the reaction volume was supplemented by nuclease free water. The cycling conditions were 40 s at 94 °C, 40 s at 50 °C, 1 mn at 72 °C for 35 cycles. Secondary PCRs were performed using 50 µl reactions containing 25 µl of GoTaq^®^ Hot start, 1 µl of each Plas3/Plas4 primers, and 1 µl of the primary PCR product completed with nuclease free water. The cycling conditions were 40 s at 94 °C, 40 s at 50 °C, 1 mn at 72 °C, and a final extension at 72 °C for 7 mn. All PCR reactions were preceded by an initial denaturation at 94 °C for 5 mn. PCR products were visualized in an electrophoresis gel and sent to Genoscreen (Lille, France) for direct Sanger sequencing on both strands using forward and reverse primers.

### Phylogenetic analyses

Sequences from positive individual of bats and nycteribiid flies were visually edited in *Geneious* 6.1.4 (http://www.geneious.com/) [37]. For positive individuals, the consensus nucleotide sequences from each were saved and used in the phylogenetic study. For similar consensus nucleotide sequences obtained from bats, the different haplotype sequences were identified using “pegas” package [38] implemented in R software [39] and only one sequence per haplotype was used in the analysis (Additional file 1: Table S1). Selected nucleotide sequences were then combined with those downloaded from GenBank and subsequently aligned using MAFFT implemented in *Geneious* software [37].

Prior to conducting phylogenetic analyses, a jModelTest version 2.1.3 [40, 41] was performed revealing GTR + G as the best substitution model. Subsequently, Bayesian inference consisting of two independent runs of four incremental Metropolis Coupled Markov Chain Monte Carlo (MC^3^) iterations starting from a random tree was conducted using MrBayes 3.1.2 [42]. This analysis was run for 5,000,000 generations with trees and associated model parameters sampled every 500 generations. The first 10% of the trees were discarded as a burn-in. New nucleotide sequences produced in the context of this study were deposited in GenBank under accession numbers MH744503 to MH744537 (Additional file 1: Table S1).

### Morphological examination of blood parasites

In the field, a thin blood smear was prepared from each captured bat, specifically to examine haemoparasite morphology. Blood smears were fixed with methanol and stained with GIEMSA at room temperature. In the context of this study, blood smears were not used for detecting infection, as PCR analysis is distinctly more sensitive than visual inspection [17, 43–45]. Further, in many cases, morphology of apicomplexan parasites does not provide sufficient characters for species-level identification. Nevertheless, some blood smears of PCR positive samples were examined to document the morphology of associated haemosporidian parasites for illustrative purposes. In such cases, blood smears were examined using a binocular microscope under immersion oil objective with 1000× magnification.

### Statistical analyses

Generalized Linear Model (GLM) analyses were conducted to investigate variation in *Polychromophilus* infection rate between species and sex classes in *Miniopterus* spp. and a Chi square analysis was conducted to compare infection rates between sex classes in *M. goudoti*. All statistical computations were conducted using R version 3.0.0 [39].

## Results

In total, 947 individual bats belonging to 31 of the 46 species currently recognized taxa on Madagascar were tested for the presence of *Polychromophilus* spp. Three of the seven tested families—Miniopteridae, Vespertilionidae and Rhinonycteridae—were found positive based on molecular screening with a total infection rate of 13.5% (Table 1). Within the Miniopteridae, all tested species were positive with significant difference in infection rates between species, ranging from 25.8% in *Miniopterus mahafaliensis* to 72.7% in *M. gleni*. Further, males had a greater chance of being infected than females (Table 2). Within the Vespertilionidae, only *M. goudoti* was found positive and had an infection rate of 41.7%; the difference in infection rates between males and females in *M. goudoti* was not statistically significant (Pearson Chi square: X^2^ = 1.62, d.f. = 1, P = 0.203). Finally, within the Rhinonycteridae, only a single individual of *P. furculus* (7.1%) tested positive for *Polychromophilus*.

**Table 1:** Infection rates of *Polychromophilus* spp. in Malagasy bats based on molecular screening

Family	Species	Tested	Positive	Negative	IR	TI
Pteropodidae	*Pteropus rufus*	20	0	20	0	0
	*Eidolon dupreanum*	11	0	11	0	0
	*Rousettus madagascariensis*	49	0	49	0	0
Hipposideridae	*Hipposideros commersoni*	27	0	27	0	0
Rhinonycteridae	*Paratriaenops furculus*	14	1	13	7.1	0.1
	*Triaenops menamena*	42	0	42	0	0
Emballonuridae	*Coleura kibomalandy*	3	0	3	0	0
	*Paremballonura tiavato*	6	0	6	0	0
Molossidae	*Chaerephon atsinanana*	34	0	34	0	0
	*Chaerephon leucogaster*	94	0	94	0	0
	*Mops leucostigma*	68	0	68	0	0
	*Mops midas*	19	0	19	0	0
	*Mormopterus jugularis*	152	0	152	0	0
	*Otomops madagascariensis*	39	0	39	0	0
Miniopteridae	*Miniopterus aelleni*	7	5	2	71.4	0.5
	*Miniopterus* cf. *manavi*	19	7	12	36.8	0.7
	*Miniopterus gleni*	22	16	6	72.7	1.7
	*Miniopterus griffithsi*	7	5	2	71.4	0.5
	*Miniopterus griveaudi*	116	43	73	37.1	4.5
	*Miniopterus mahafaliensis*	89	23	66	25.8	2.4
	*Miniopterus majori*	7	2	5	28.6	0.2
	*Miniopterus sororculus*	22	8	14	36.4	0.8
Vespertilionidae	*Hypsugo bemainty*	2	0	2	0	0
	*Myotis goudoti*	48	20	28	41.7	2.1
	*Neoromicia malagasyensis*	2	0	2	0	0
	*Neoromicia matroka*	3	0	3	0	0
	*Neoromicia robertsi*	2	0	2	0	0
	*Pipistrellus hesperidus*	11	0	11	0	0
	*Pipistrellus raceyi*	3	0	3	0	0
	*Pipistrellus/Neoromicia* sp.	8	0	8	0	0
	*Scotophilus marovaza*	1	0	1	0	0
	Total	947	130	817		13.5

*IR* Infection rate per species, *TI* Total infection rates

**Table 2:** Logistic regression showing the infection risk in Malagasy *Miniopterus* spp. for *Polychromophilus melanipherus* based on species and sex

	Crude odd ratio (95% CI)	Adjusted odd ratio (95% CI)	P (Wald’s test)
Species: ref. = *Miniopterus mahafaliensis*			
*Miniopterus aelleni*	7.17 (1.3, 39.55)	9.29 (1.62, 53.4)	0.012
*Miniopterus manavi* sensu lato	1.67 (0.59, 4.76)	1.51 (0.53, 4.32)	0.44
*Miniopterus gleni*	7.65 (2.67, 21.9)	10.14 (3.39, 30.35)	< 0.001
*Miniopterus griffithsi*	7.17 (1.3, 39.55)	8.27 (1.46, 46.93)	0.017
*Miniopterus griveaudi*	1.69 (0.92, 3.1)	2.51 (1.26, 4.99)	0.009
*Miniopterus majori*	1.15 (0.21, 6.33)	1.4 (0.25, 7.92)	0.706
*Miniopterus sororculus*	1.64 (0.61, 4.41)	2.75 (0.93, 8.14)	0.067
Sex: male vs female	1.43 (0.88, 2.32)	2.16 (1.18, 3.94)	0.012

### Phylogenetic analyses and host-parasite relationship

**Fig. 2:**
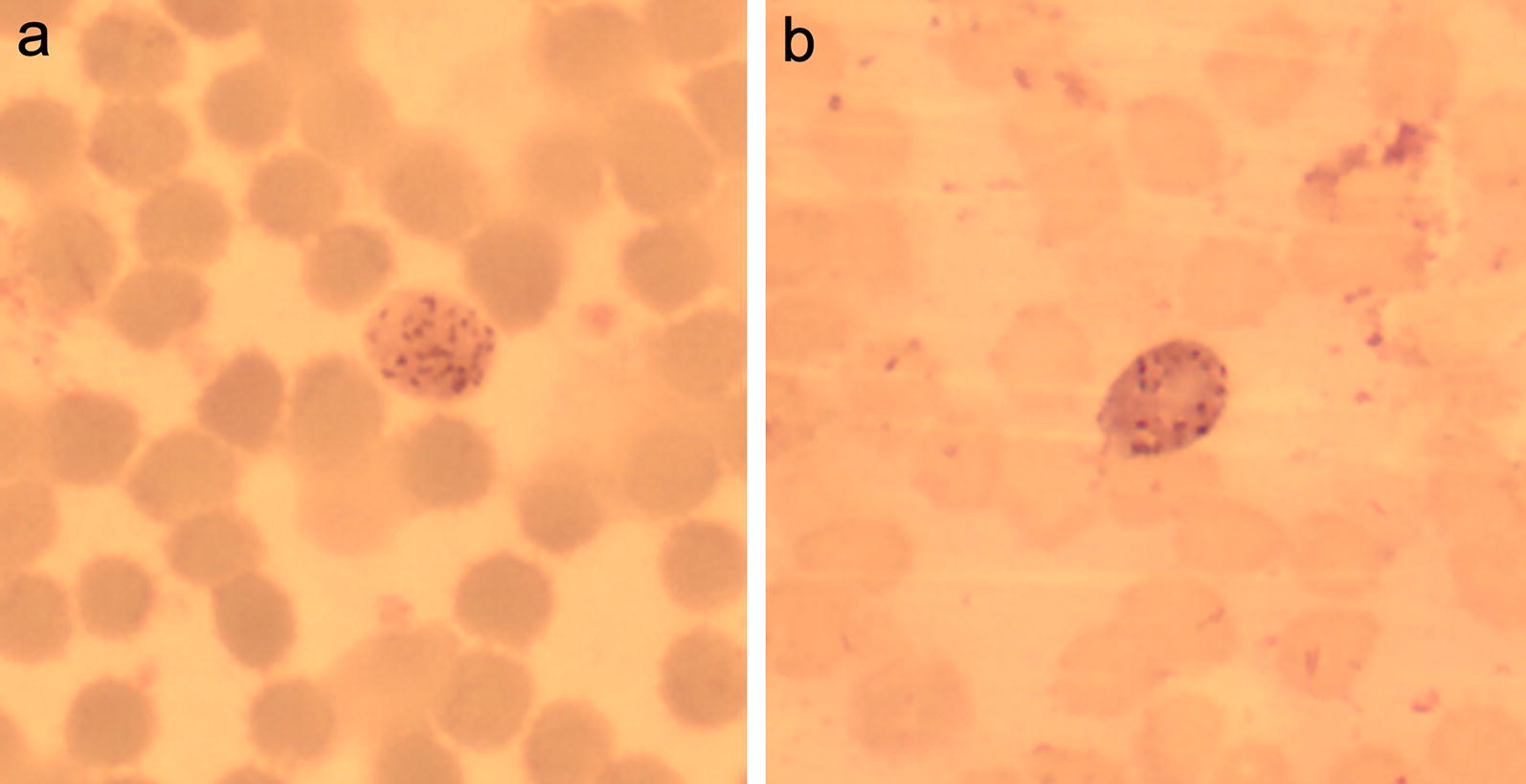
Representative micrograph of *Polychromophilus melanipherus* and *Polychromophilus murinus* gametocytes from Giemsa-stained blood smears. **a** *Polychromophilus melanipherus* infecting *Miniopterus majori* and **b** *Polychromophilus murinus* infecting *Myotis goudoti*

**Fig. 3:**
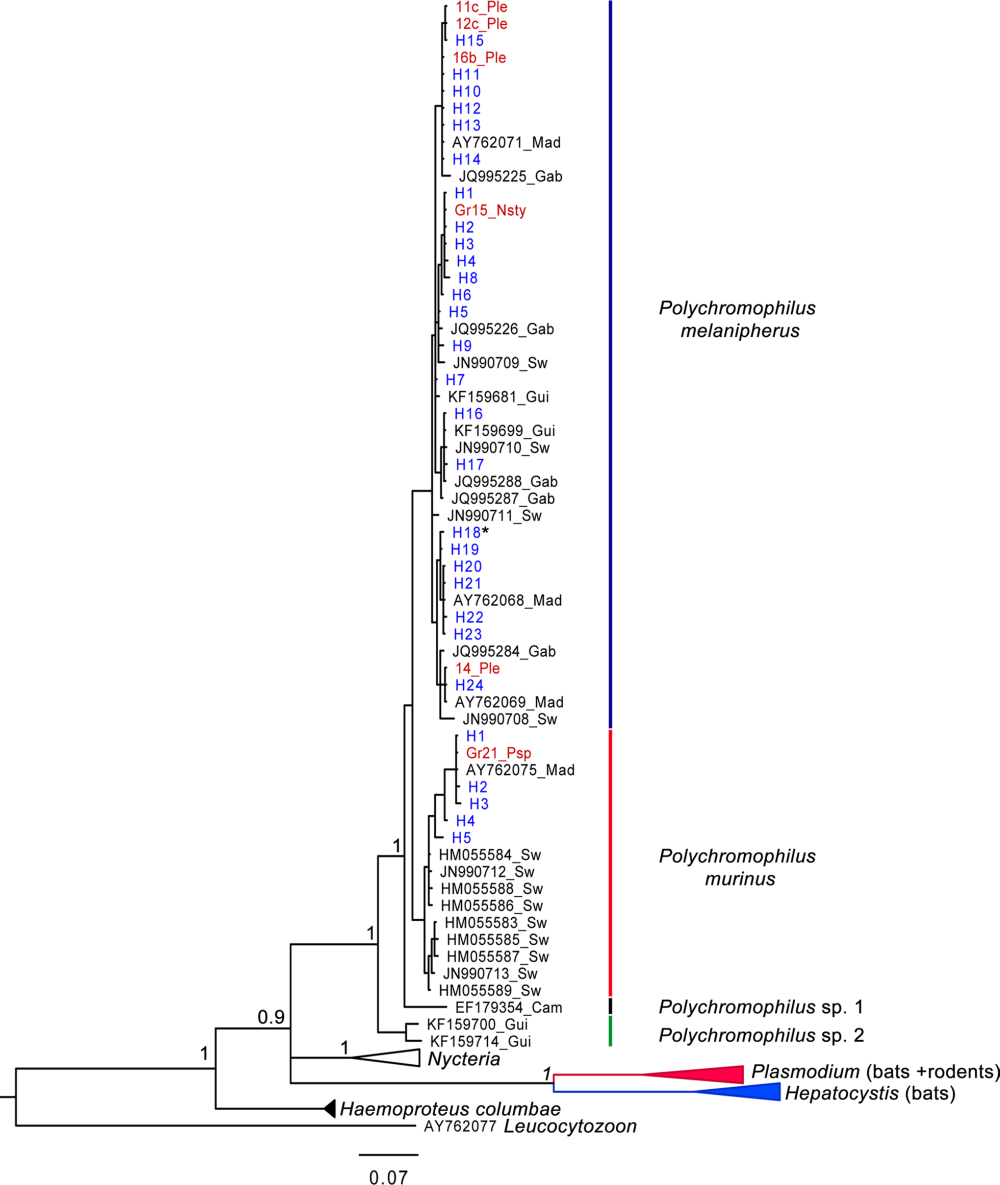
Bayesian reconstruction showing *Polychromophilus* spp. infecting Malagasy bats (in blue) and Nycteribiidae (in red) based on cytochrome *b*. Only values in the major nodes were represented for higher posterior probabilities (> 0.9). *Polychromophilus melanipherus* identified in *Paratriaenops furculus* are followed by an asterisk. Mad: Madagascar, Gui: Guinea, Sw: Switzerland, Gab: Gabon, Ple: *Penicillidia leptothrinax*, Psp: *Penicillidia* sp. (cf. *fulvida*), Nsty: *Nycteribia stylidiopsis*

Phylogenetic analysis based on *cyt b* revealed that *Polychromophilus* infecting bats in different portion of the world forms a monophyletic clade composed of four clusters. *Polychromophilus* in Malagasy bats is segregated into two clusters. A first cluster is composed of *P. melanipherus* (Fig. 2a) lineages infecting all eight *Miniopterus* spp. from Madagascar screened in the present investigation, as well as a single individual of *P. furculus* (H1 to H24 in the phylogenetic tree, Fig. 3, Additional file 1: Table S1). *Polychromophilus melanipherus* identified from Malagasy Miniopteridae clustered with those identified in *M. schreibersii* from Switzerland, *Miniopterus inflatus* from Gabon, and *Miniopterus villiersi* from Guinea (Fig. 3). Hence, excluding *P. furculus*, *P. melanipherus* is known from the Miniopteridae of Africa, Europe, and Madagascar. The second cluster is composed of *P. murinus* (Fig. 2b) recovered from *M. goudoti* on Madagascar (H1 to H5 in the phylogenetic tree, Fig. 3, Additional file 1: Table S1). Although the Malagasy clade of *P. murinus* forms a monophyletic group with haemoparasites recovered from *Myotis daubentonii* and other European Vespertilionidae (Fig. 3), they are not embedded within the clade and show a certain level of genetic divergence. Haemosporidian parasites identified from *Kerivoula hardwickii* (Vespertilionidae) sampled in Cambodia and from *Neoromicia capensis* and *Pipistrellus* aff. *grandidieri* (Vespertilionidae) sampled in Guinea cluster in two distinct clades labelled *Polychromophilus* sp. 1 and 2, respectively (Fig. 3).

### Molecular screening of Nycteribiidae: insights on their role as potential vectors

In total, 38 individual nycteribiids belonging to three species (17 *Penicillidia leptothrinax*, 2 *Penicillidia* sp. (cf. *fulvida*), and 19 *Nycteribia stylidiopsis*) were tested via PCR screening for the presence of *Polychromophilus*. Five bat fly specimens were positive for *P. melanipherus* and one for *P. murinus*. Bat flies positive for *P. melanipherus* via the PCR analyses included four *P. leptothrinax* (two sampled on *Miniopterus aelleni* and two on *M. manavi* s.l.) in addition to a *N. stylidiopsis* obtained on *M. gleni*. Of note, this latter nycteribiid was sampled on a PCR negative bat host. Further, one *Penicillidia* sp. (cf. *fulvida*) specimen collected on *Miniopterus griveaudi* was found positive for *P. murinus*, whereas the associated bat host was infected with *P. melanipherus*.

## Discussion

Malarial parasites are known to infect a wide diversity of vertebrates, specifically birds [43, 45, 46], reptiles [1, 47], and mammals [1], including bats [6, 17, 19]. This paraphyletic apicomplexan group is highly diverse with different speciation and host-switching events [1, 8]. Haemosporidian parasites infecting bats were first reported by Dionisi in 1899 [20] and an updated checklist of chiropteran haemosporidia has been recently published based on their gametocyte types and schizont locations [16]. With respect to Malagasy bats, a few studies have been conducted to detect and identify haemosporidian parasites in these animals based on morphological and molecular tools [8, 28]. However, a number of bat taxa have hitherto not been screened; a necessary step to understand infection patterns and identify biotic and abiotic parameters influencing parasite transmission.

### Phylogenetic relationship of haemosporidian parasites

Previous phylogenetic studies of haemosporidian parasites infecting Malagasy bats revealed the presence of two different parasite species belonging to the genus *Polychromophilus* [6, 8, 17]. In this study, the presence of two *Polychromophilus* sister species infecting two widely distributed bat genera on Madagascar, namely *Miniopterus* (Miniopteridae) and *Myotis* (Vespertilionidae) was confirmed. *Polychromophilus melanipherus* infects all eight *Miniopterus* spp. sampled in the present study, which are endemic to the Malagasy Region (Madagascar and the Comoros). The haplotypes of haemoparasites infecting Malagasy bats are embedded within the *P. melanipherus* clade occurring in African and European *Miniopterus* spp. This topology strongly supports a tight specificity of *P. melanipherus* within hosts belonging to the Miniopteridae [6, 17, 48]. One individual of *P. furculus* (Rhinonycteridae) also tested positive and based on the *cyt b* sequence, it fell within the *P. melanipherus* clade. Raharimanga and colleagues [28] and Duval and colleagues [8], already reported the presence of one individual of *P. furculus* with haemosporidian infection based on microscopic examination. This *P. furculus* specimen might have been accidentally and transiently infected by an infected nycteribiid during a blood meal. The haplotype identified in this *P. furculus* is unique and different from haplotypes obtained from infected *Miniopterus* sampled in the same day roost. However, further investigations are needed to elucidate the importance of *P. melanipherus* infection in *P. furculus* with a greater sample from different localities.

The second cluster of *Polychromophilus* only infected *M. goudoti* and is genetically related to *P. murinus*. Interestingly, *P. murinus* from *M. goudoti*, a bat endemic to Madagascar, was not embedded within the cluster of *P. murinus* from European bat species and suggesting a different diversification history of these two subgroups. It has been suggested that the lineage in which *M. goudoti* is placed (Ethiopian clade V) diverged from the other lineages mainly composed of Palearctic and Oriental *Myotis* spp. (Clade I, II, III, IV) in the Miocene, over 11 Mya ago [49]. It can thus be hypothesized that *P. murinus* parasites infecting *M. goudoti* co-diverged with their host and infects a higher diversity of bats within the family Vespertilionidae such as *Myotis daubentonii*, *Eptesicus serotinus*, and *Nyctalus noctula* [8, 18].

No co-infection was found in the present study, which confirms previously published information [6, 17]. This suggests that infection is actually specific at the level of host genus. This pattern is remarkable in that on Madagascar *Miniopterus* spp. and *M. goudoti* are syntopic in the same day-roost sites. Such physical contact has been previously reported to favour host switching of pathogenic *Leptospira* between these two syntopic occurring host genera [32], but this does not seem to be the case for *Polychromophilus* spp.

### *Polychromophilus* infection in bats from Madagascar

The molecular screening of Malagasy bats revealed a total infection rate of 13.5%, which is congruent with previously reported rates [8, 28]. Based on the analysis of 31 Malagasy bat taxa, haemosporidian infection is limited to the families Miniopteridae, Vespertilionidae, and Rhinonycteridae. Bayesian reconstruction showed the presence of *P. melanipherus* infecting all eight *Miniopterus* spp. sampled herein, as well as a single *P. furculus* specimen, while *P. murinus* was only detected in *M. goudoti*. While a significant difference was observed between sexes in *P. melanipherus* infecting *Miniopterus* spp., no such pattern was observed in *P. murinus* infecting *M. goudoti*. These results can be explained by the roosting ecology and behaviour of these two syntopic species. Both sexes of *M. goudoti* live together in colonies throughout the year. For *Miniopterus* spp., there is at least partial sexual segregation in day-roosting sites. For example, *M. manavi* s.l. was sampled on several occasions in an open rock-shelter cave at Ambohitantely, where only males were present, suggesting that the two sexes are in contact only during the initial stages of reproduction. Further, bats roosting in this shallow cave are presumably more exposed to haematophagous insects than bats of either sex roosting deep in caves. Nevertheless, for species such as *M. mahafaliensis* sampled in the present study and at different localities, both sexes were present within their day roost site.

### Insight into invertebrate vectors

*Polychromophilus* spp. have been reported to be transmitted by nycteribiid flies [13, 14, 50], wingless Diptera that are obligate blood-sucking parasites of bats [34]. Molecular screening of bat flies sampled in the context of this study was carried out to identify potential candidate vectors. The screening results should be interpreted with some caution, as the presence of *Polychromophilus* DNA in a nycteribiid might simply be the result from a recent blood meal. Flies sample included *P. leptothrinax*, specific to *Miniopterus* spp., and *Penicillidia* sp. (cf. *fulvida*) and *N. stylidiopsis*, parasitizing both *Myotis* and *Miniopterus* [30, 33]. In several cases, *Penicillidia* sp. (cf. *fulvida*) and *N. stylidiopsis* were found on the same bat host [30, 33]. Both *P. leptothrinax* and *N. stylidiopsis* tested positive for *P. melanipherus*. Further, *P. murinus* was detected in a *Penicillidia* sp. (cf. *fulvida*) fly that was collected on a *Miniopterus griveaudi* specimen testing positive for *P. melanipherus*. Although, cross contamination cannot be excluded during bat sampling as *Miniopterus griveaudi* and *M. goudoti* occur in syntopic cave day-roost sites, the result can alternatively be interpreted as additional evidence for *Miniopterus* spp. being non-permissive to *P. murinus* infection. This situation may be related to the ecology of *Miniopterus* and *Myotis*. In fact, colonies of *Miniopterus* spp. and *M. goudoti* often live in syntopy (physical contact). Members of these two genera seem to have ectoparasites with relaxed host preference [30, 33]. However, given their close physical contact, it might be expected to favor parasite co-infection and/or host switching. However, no *P. murinus/P. melanipherus* co-infection was detected either in bat genera or in their nycteribiid ectoparasites [6, 17, 51], and thus suggesting host-specificity.

## Conclusions

This work provides further advances regarding previous studies on the taxonomy and distribution of *Polychromophilus* spp. in bats occurring on Madagascar. While *Polychromophilus* infections seem to be mostly limited to the families Miniopteridae and Vespertilionidae, the presence of other apicomplexan parasites, such as *Plasmodium* and *Hepatocystis,* should also be investigated using molecular and morphological techniques. As a vector-borne infection, future work on haemosporidian parasites should carefully address the biology, ecology, and distribution of invertebrate vectors. The important diversity of Malagasy bats, especially the 12 currently recognized species of *Miniopterus* with different distributions and reproduction behaviour, together with the specificity of *Polychromophilus* make this biological model particularly suitable to investigate the impact of biotic and abiotic factors on the transmission of haemoparasites.

## Additional file

**Additional file 1: Table S1.** Parasites included in the present study, including Haplotype, Isolate, GenBank accession numbers, host species, Museum voucher and origin. Molecular data produced in the frame of the present work are marked with an asterisk (*). FMNH = Field Museum of Natural History, UADBA = Université d’Antananarivo, Département de Biologie Animale, NA: not available.
