# Supplementary material for: Polychromophilus spp. (Haemosporida) in Malagasy bats: host specificity and insights on invertebrate vectors
Source: Malar J. 2018 Aug 31;17:318. doi: 10.1186/s12936-018-2461-8 (PMC6119311; doi:10.1186/s12936-018-2461-8)
Supplement: Supplementary file 1 — Additional file 1: Table S1. Parasites included in the present study, including Haplotype, Isolate, GenBank accession numbers, host species, Museum voucher and origin. Molecular data produced in the frame of the present work are marked with an asterisk (*). FMNH = Field Museum of Natural History, UADBA = Université d’Antananarivo, Département de Biologie Animale, NA: not available. [file 12936_2018_2461_MOESM1_ESM.docx]

Table S1. Parasites included in the present study, including Haplotype, Isolate, GenBank accession numbers, host species, Museum voucher and origin. Molecular data produced in the frame of the present work are marked with an asterisk (*). FMNH = Field Museum of Natural History, UADBA = Université d’Antananarivo, Département de Biologie Animale, NA: not available

| **Parasite** | **Haplotypes** | **Isolate** | **Genbank** | **Host species** | **Groups** | **Museum** | **Origin** |
| --- | --- | --- | --- | --- | --- | --- | --- |
|  |  |  | **Number** |  |  | **voucher** |  |
| *P. melanipherus* | H1 (n=18) | 36MG | MH744503 | *Miniopterus mahafaliensis* | Bat | FMNH 217957 | Madagascar |
| *P. melanipherus* | H2 (n=1) | 744MG | MH744504 | *Miniopterus mahafaliensis* | Bat | FMNH 218041 | Madagascar |
| *P. melanipherus* | H3 (n=1) | 754MG | MH744505 | *Miniopterus mahafaliensis* | Bat | UADBA SMG 17344 | Madagascar |
| *P. melanipherus* | H4 (n=1) | 169MG | MH744506 | *Miniopterus griffithsi* | Bat | UADBA SMG 17447 | Madagascar |
| *P. melanipherus* | H5 (n=6) | 75MG | MH744507 | *Miniopterus mahafaliensis* | Bat | UADBA 32354 | Madagascar |
| *P. melanipherus* | H6 (n=3) | 545MG | MH744508 | *Miniopterus griveaudi* | Bat | FMNH 221345 | Madagascar |
| *P. melanipherus* | H7 (n=1) | 775MG | MH744509 | *Miniopterus gleni* | Bat | UADBA 50170 | Madagascar |
| *P. melanipherus* | H8 (n=1) | 781MG | MH744510 | *Miniopterus gleni* | Bat | FMNH 218032 | Madagascar |
| *P. melanipherus* | H9 (n=1) | 583MG | MH744511 | *Miniopterus gleni* | Bat | UADBA 33027 | Madagascar |
| *P. melanipherus* | H10 (n=1) | 51MG | MH744512 | *Miniopterus manavi* sensu lato | Bat | FMNH 221423 | Madagascar |
| *P. melanipherus* | H11 (n=8) | 53MG | MH744513 | *Miniopterus manavi* sensu lato | Bat | FMNH 221429 | Madagascar |
| *P. melanipherus* | H12 (n=1) | 437MG | MH744514 | *Miniopterus griveaudi* | Bat | UADBA 33962 | Madagascar |
| *P. melanipherus* | H13 (n=11) | 502MG | MH744515 | *Miniopterus griveaudi* | Bat | FMNH 221338 | Madagascar |
| *P. melanipherus* | H14 (n=1) | 547MG | MH744516 | *Miniopterus griveaudi* | Bat | FMNH 221347 | Madagascar |
| *P. melanipherus* | H15 (n=5) | 601MG | MH744517 | *Miniopterus griveaudi* | Bat | FMNH 221350 | Madagascar |
| *P. melanipherus* | H16 (n=11) | 38MG | MH744518 | *Miniopterus gleni* | Bat | UADBA SMG 17171 | Madagascar |
| *P. melanipherus* | H17 (n=1) | 183MG | MH744519 | *Miniopterus griffithsi* | Bat | UADBA SMG 17464 | Madagascar |
| *P. melanipherus* | H18 (n=3) | 57MG | MH744520 | *Paratriaenops furculus* | Bat | UADBA SMG 17186 | Madagascar |
| *P. melanipherus* | H19 (n=2) | 584MG | MH744521 | *Miniopterus gleni* | Bat | UADBA 33028 | Madagascar |
| *P. melanipherus* | H20 (n=22) | 318MG | MH744522 | *Miniopterus griveaudi* | Bat | UADBA 32938 | Madagascar |
| *P. melanipherus* | H21 (n=1) | 341MG | MH744523 | *Miniopterus griveaudi* | Bat | UADBA 32954 | Madagascar |
| *P. melanipherus* | H22 (n=1) | 518MG | MH744524 | *Miniopterus griveaudi* | Bat | UADBA 33014 | Madagascar |
| *P. melanipherus* | H23 (n=1) | 513MG | MH744525 | *Miniopterus griveaudi* | Bat | UADBA 33009 | Madagascar |
| *P. melanipherus* | H24 (n=8) | 52MG | MH744526 | *Miniopterus manavi* sensu lato | Bat | FMNH 221425 | Madagascar |
| *P. melanipherus* |  | **Gr15** | MH744527 | ***Nycteribia stylidiopsis*** | Nycteribiidae |  | Madagascar |
| *P. melanipherus* |  | **14** | MH744528 | ***Penicillidia leptothrinax*** | Nycteribiidae |  | Madagascar |
| *P. melanipherus* |  | **16b** | MH744529 | ***Penicillidia leptothrinax*** | Nycteribiidae |  | Madagascar |
| *P. melanipherus* |  | **11c** | MH744530 | ***Penicillidia leptothrinax*** | Nycteribiidae | FMNH 221440 | Madagascar |
| *P. melanipherus* |  | **12c** | MH744531 | ***Penicillidia leptothrinax*** | Nycteribiidae |  | Madagascar |
| *P. melanipherus* |  |  | JN990708 | *Miniopterus schreibersii* | Bat |  | Switzerland |
| *P. melanipherus* |  |  | JN990709 | *Miniopterus schreibersii* | Bat |  | Switzerland |
| *P. melanipherus* |  |  | JN990710 | *Miniopterus schreibersii* | Bat |  | Switzerland |
| *P. melanipherus* |  |  | JN990711 | *Miniopterus schreibersii* | Bat |  | Switzerland |
| *P.* sp. |  |  | KF159699 | *Miniopterus villiersi* | Bat |  | Guinea |
| *P.* sp. |  |  | KF159681 | *Miniopterus villiersi* | Bat |  | Guinea |
| *P. melanipherus* |  |  | AY762071 | *Miniopterus griveaudi* | Bat | FMNH 172862 | Madagascar |
| *Haemosporida* sp. |  |  | AY762068 | *Miniopterus griveaudi* | Bat | FMNH 172831 | Madagascar |
| *Haemosporida* sp. |  |  | AY762069 | *Miniopterus mahafaliensis* | Bat | FMNH 172924 | Madagascar |
| *P.* sp. |  |  | JQ995285 | *Miniopterus inflatus* | Bat |  | Gabon |
| *P.* sp. |  |  | JQ995284 | *Miniopterus inflatus* | Bat |  | Gabon |
| *P.* sp. |  |  | JQ995286 | *Miniopterus inflatus* | Bat |  | Gabon |
| *P*. sp. |  |  | JQ995287 | *Miniopterus inflatus* | Bat |  | Gabon |
| *P.* sp. |  |  | JQ995288 | *Miniopterus inflatus* | Bat |  | Gabon |
| *P. murinus* | H1 (n=12) | 47MG | MH744532 | *Myotis goudoti* | Bat | FMNH 217967 | Madagascar |
| *P. murinus* | H2 (n=1) | 493MG | MH744533 | *Myotis goudoti* | Bat | FMNH 221321 | Madagascar |
| *P. murinus* | H3 (n=2) | 594MG | MH744534 | *Myotis goudoti* | Bat | UABBA 33001 | Madagascar |
| *P. murinus* | H4 (n=1) | 722MG | MH744535 | *Myotis goudoti* | Bat | FMNH 218006 | Madagascar |
| *P. murinus* | H5 (n=4) | 554MG | MH744536 | *Myotis goudoti* | Bat | UADBA 32995 | Madagascar |
| *P. murinus* |  | **Gr21** | MH744537 | *Penicillidia* sp. | Nycteribiidae |  | Madagascar |
| *P. murinus* |  |  | HM055583 | Not specified | Bat |  | Switzerland |
| *P. murinus* |  |  | HM055584 | Not specified | Bat |  | Switzerland |
| *P. murinus* |  |  | HM055585 | Not specified | Bat |  | Switzerland |
| *P. murinus* |  |  | HM055586 | Not specified | Bat |  | Switzerland |
| *P. murinus* |  |  | HM055587 | Not specified | Bat |  | Switzerland |
| *P. murinus* |  |  | HM055588 | Not specified | Bat |  | Switzerland |
| *P. murinus* |  |  | HM055589 | Not specified | Bat |  | Switzerland |
| *P. murinus* |  |  | JN990712 | *Myotis daubentonii* | Bat |  | Switzerland |
| *P. murinus* |  |  | JN990713 | *Myotis daubentonii* | Bat |  | Switzerland |
| *P. murinus* |  |  | AY762075 | *Myotis goudoti* | Bat | FMNH 175810 | Madagascar |
| *P.* sp. 2 |  |  | KF159714 | *Pipistrellus grandidieri* | Bat |  | Guinea |
| *P.* sp. 2 |  |  | KF159700 | *Neoromicia capensis* | Bat |  | Guinea |
| *Hepatocystis* sp. |  |  | KF188066 | *Myonycteris leptodon* | Bat |  | Côte d'Ivoire |
| *Hepatocystis* sp. |  |  | KF159691 | *Micropteropus pusillus* | Bat |  | Guinea |
| *Hepatocystis* sp. |  |  | KF159696 | *Micropteropus pusillus* | Bat |  | Guinea |
| *Hepatocystis* sp. |  |  | KF159684 | *Nanonycteris veldkampii* | Bat |  | Côte d'Ivoire |
| *Hepatocystis* sp. |  |  | KF159715 | *Nanonycteris veldkampii* | Bat |  | Côte d'Ivoire |
| *Hepatocystis* sp. |  |  | KF159705 | *Myonycteris leptodon* | Bat |  | Liberia |
| *Hepatocystis* sp. |  |  | KF159683 | *Micropteropus pusillus* | Bat |  | Guinea |
| *Hepatocystis* sp. |  |  | KF159698 | *Nanonycteris veldkampii* | Bat |  | Liberia |
| *Hepatocystis* sp. |  |  | KF159676 | *Micropteropus pusillus* | Bat |  | Guinea |
| *P.* sp.1 |  |  | EF179354 | *Kerivoula* sp. | Bat |  | Cambodia |
| *Nycteria* sp. |  |  | EF179355 | *Megaderma spasma* | Bat |  | Cambodia |
| *Hepatocystis* sp. |  |  | EF179356 | *Hipposideros larvatus* | Bat |  | Cambodia |
| *Nycteria* sp. |  |  | KF159720 | *Rhinolophus alcyone* | Bat |  | Côte d'Ivoire |
| *Nycteria* sp. |  |  | KF159690 | *Rhinolophus landeri* | Bat |  | Guinea |
| *Plasmodium chabaudi* |  |  | AY099050 | *Thamnomys rutilans* | Rodent |  | Central African Republic |
| *Plasmodium yoelii* |  |  | AY099051 | *Thamnomys rutilans* | Rodent |  | Central African Republic |
| *Plasmodium berghei* |  |  | DQ414645 | *Grammomys surdaster* | Rodent |  | Democratic Republic of the Congo |
| *Plasmodium vinckei* |  |  | DQ414654 | *Thamnomys rutilans* | Rodent |  | Democratic Republic of the Congo |
| *Plasmodium vinckei* |  |  | DQ414655 | *Thamnomys rutilans* | Rodent |  | Central African Republic |
| *Plasmodium vinckei* |  |  | DQ414656 | *Thamnomys rutilans* | Rodent |  | Central African Republic |
| *Plasmodium voltaicum* |  |  | KF159671 | *Myonycteris angolensis* | Bat |  | Guinea |
| *Plasmodium cyclopsi* |  |  | KF159710 | *Hipposideros cyclops* | Bat |  | Liberia |
| *Plasmodium cyclopsi* |  |  | KF159716 | *Hipposideros cyclops* | Bat |  | Liberia |
| *Plasmodium cyclopsi* |  |  | KF159674 | *Hipposideros cyclops* | Bat |  | Liberia |
| *Haemoproteus columbae* |  |  | EU254548 | *Columba livia* | Bird |  | USA |
| *Haemoproteus columbae* |  |  | EU254553 | *Columba livia* | Bird |  | USA |
| *Leucocytozoon* sp. |  |  | AY762077 | Not specified | Bird |  | Madagascar |
